# Association of *DPYD* rs4294451, plasma uracil concentration, and sex with 5-fluorouracil exposure in patients with gastrointestinal cancer

**DOI:** 10.1007/s00280-025-04851-z

**Published:** 2025-12-04

**Authors:** Gabriel A. Brooks, Dylan B. Ness, Kathryn C. Hourdequin, Gregory H. Ripple, Manik Amin, Sierra Lord-Halvorson, Wahab A. Khan, Sophie J. Deharvengt, Vincent Busque, Konstantin H. Dragnev, Wenyan Zhao, Tor D. Tosteson, Lionel D. Lewis

**Affiliations:** 1 Dartmouth Cancer Center, Lebanon, NH USA; 2 Geisel School of Medicine, Lebanon, NH USA; 3 Department of Medicine, Dartmouth Hitchcock Medical Center, 1 Medical Center Drive, Lebanon, NH 03756 USA; 4 University of Chicago Comprehensive Cancer Center, Chicago, IL USA

**Keywords:** 5-fluorouracil, Gastrointestinal cancer, Uracil, DPYD

## Abstract

**Purpose:**

Standard dosing of infusional 5-FU results in subtherapeutic drug exposure for up to half of all patients. We evaluated plasma uracil concentration and *DPYD* rs4294451 genotype as candidate predictors of individual-level 5-FU exposure.

**Methods:**

We conducted a prospective study of drug exposure in patients with gastrointestinal cancer receiving infusional 5-FU. Participants were evaluated by measurement of fasting pretreatment plasma uracil concentration, *DPYD* gene sequence (including genotyping of rs4294451) and 5-FU area under the curve (5-FU AUC). We used a linear mixed-effects model to evaluate the association of 5-FU AUC with uracil concentration and rs4294451 genotype, adjusting for cycle number, sex, serum creatinine, and 5-FU dose.

**Results:**

There were 29 evaluable participants with a median age of 64 years (range 41–81); nine (31%) were female. The median plasma uracil concentration was 10.4 ng/mL (IQR 7.2, 12.6). Nine participants carried the *DPYD* rs4294451 T-allele (7 with T/A, 2 with T/T.) Among all participants the median 5-FU AUC was 22.0 mg*h/L in cycle 1 and 19.3 mg*h/L in cycle 2. In the mixed-effects model, higher rs4294451 T-allele count (0, 1, or 2) was significantly associated with lower 5-FU AUC (-4.0 mg*h/L per T-allele [95% CI -8.0, 0.0], *p* = 0.049), as was male sex (-7.5 mg*h/L [95% CI -13.8, -1.3], *p* = 0.021). Pretreatment plasma uracil concentration was not significantly associated with 5-FU AUC (*p* = 0.57). The subject with the highest uracil concentration (23.1 ng/mL) had a rare *DPYD* missense variant (c.2185G > A [p.A729T]) and experienced early 5-FU-related toxicity.

**Conclusions:**

*DPYD* rs4294451 T-allele count and male sex were significantly associated with reduced 5-FU drug exposure. *DPYD* rs4294451 and male sex merit further evaluation as candidate biomarkers to inform initial dosing of infusional 5-FU.

## Introduction

5-fluorouracil (5-FU) has been one of the most essential systemic therapies for solid tumors for more than a half century, and 5-FU continues to play a critical role in the treatment of many cancers [1–3]. The modes of 5-FU administration have evolved over the years; initial regimens were based on bolus administration, but modern fluorouracil-based regimens (e.g. mFOLFOX6 and mFOLFIRINOX) rely heavily on infusional administration, most commonly as a 46 h infusion repeated every 14 days [4]. The transition from bolus to infusional regimens has resulted in preserved or enhanced treatment efficacy with reduced treatment-related toxicity – a win-win outcome for patients with cancer. Still, toxicities from 5-FU (including mucositis, diarrhea, neutropenia, and infection) remain common, and can sometimes result in hospitalization or even death [5, 6].

The narrow therapeutic index of 5-FU requires that treatment protocols navigate carefully between the opposing hazards of over- and under-dosing. Unlike severe toxicity from 5-FU, subtherapeutic exposure is not directly observable in routine clinical care. However, subtherapeutic drug exposure is discoverable by therapeutic drug monitoring (TDM, i.e. pharmacokinetic assay of the 5-FU area under the curve [AUC]) [7–9]. Prior research on 5-FU pharmacokinetics and TDM has convincingly demonstrated substantial inter-individual variation in 5-FU AUC, with evidence for clinically relevant impacts on toxicity and treatment efficacy [7, 8, 10]. Based on studies reporting data for both drug exposure and toxicities, an expert panel has endorsed a 5-FU AUC therapeutic target range of 20–30 mg*h/L [8].

As with most other chemotherapy drugs, protocols for 5-FU dosing are applied to an individual patient based on body surface area (BSA), calculated from the patient’s height and weight. Regrettably, several studies demonstrate that more than 50% of patients do not achieve therapeutic-range exposure to infusional 5-FU with standard BSA-adjusted dosing [10–12], potentially resulting in inferior cancer treatment outcomes. Despite the evident shortcomings of BSA-based dosing for infusional 5-FU, evidence is lacking to support the use of alternative biomarkers for dose selection. In this study, we sought to evaluate two candidate biomarkers for predicting 5-FU drug exposure: pretreatment plasma uracil concentration and rs4294451 (an intronic allele in the promoter region of the *DPYD* gene).

Pretreatment plasma uracil concentration is used to screen for dihydropyrimidine dehydrogenase (DPD) deficiency in some countries, including France [13]. Uracil is an endogenous substrate of the DPD enzyme, and high plasma uracil concentration (e.g. concentration greater than 16 ng/mL) indicates decreased DPD enzyme activity [14]. Other research suggests that increased DPD activity, as measured by in vitro radioenzymatic assay, is associated with reduced chemotherapy effectiveness and impaired progression-free and overall survival in patients receiving fluoropyrimidine chemotherapy [15]. However, this radioenzymatic assay is not widely available outside of research settings, and we hypothesized that low pretreatment plasma uracil concentration could serve as a surrogate marker for increased DPD enzyme activity.

Two recent studies have identified an apparent relationship between DPD enzyme activity and rs4294451, a locus within a cis-enhancer region of the *DPYD* gene. Zhang and colleagues conducted in vitro and in vivo analyses demonstrating that the rs4294451 T-variant (27% global minor allele frequency) increases recruitment of the CEBPB transcription factor, leading to increased gene expression and differential sensitivity to 5-FU in cell culture [16]. Subsequently, De Mattia and colleagues evaluated the relationship of the rs4294451 genotype with risk of severe (grade 4–5) toxicity from fluoropyrimidine chemotherapy in a retrospective cohort of 645 patients. They found substantial reductions in hematological and all-cause toxicity among carriers of the rs4294451 T-allele [17].

The objective of this study was to evaluate fasting pretreatment plasma uracil concentration and rs4294451 genotype as candidate predictors of 5-FU exposure, with a broader goal of identifying biomarkers that could be used to identify patients at risk for sub-therapeutic 5-FU exposure under the paradigm of BSA-adjusted chemotherapy dosing.

## Materials and methods

### Ethical considerations

This research was conducted in line with the principles of The Belmont Report of 1979. Ethical approval of the study was obtained from the Dartmouth Health IRB, and all participants provided written informed consent prior to the performance of any study activities.

### Patients and major eligibility criteria

Patients were recruited from the medical oncology clinic at the Dartmouth Cancer Center. Eligible participants were adult patients with gastrointestinal cancer who were planned to receive a chemotherapy regimen including a 46-hour infusion of 5-FU at a starting dose of 2400 mg/m^2^. During the enrollment period it was the standard of care at our center to screen for deleterious variants of the *DPYD* gene prior to treatment with 5-fluorouracil, including the *2A, *13, and c.2846 A > T variants; patients who had previously tolerated standard-dose therapy with 5-FU were exempt from this screening. Study exclusion criteria included receipt of infusional 5-FU in the previous 3 months, estimated glomerular filtration rate of less than 60 mL/minute/1.73 m^2^, known deleterious germline variants of the *DPYD* gene, total bilirubin greater than 2.5 times the upper limit of normal, and liver transaminases (AST/ALT) greater than 5 times the upper limit of normal.

The study also enrolled a second cohort of participants with deleterious *DPYD* gene variants; this cohort was enrolled with the objective of confirming the sensitivity of the plasma uracil assay for identifying patients with germline *DPYD* gene variants, (evaluation of convergent validity). Participants for this second cohort were recruited from a clinical population of known *DPYD* gene variant carriers, otherwise using the same exclusion criteria as for the primary study cohort. However, participants in this cohort were not required to be initiating treatment with infusional 5-FU.

### Study objectives and outcome measures

The primary study objective was to evaluate the association of fasting pretreatment plasma uracil concentration and rs4294451 genotype with 5-FU drug exposure (as measured by the 5-FU AUC.) We performed additional analyses to evaluate the relationship of pretreatment plasma uracil concentration with less common *DPYD* gene variants and early 5-FU-related toxicity. We defined early 5-FU-related toxicity as occurrence of grade 3 + neutropenia, grade 3 + thrombocytopenia, or reduction of the 5-FU infusion dose for any reason during the first 4 cycles of chemotherapy. Toxicity grading was done according to the Common Terminology Criteria for Adverse Events, version 5.0.

### Study procedures

All study participants provided pretreatment blood samples for measurement of plasma uracil and dihydrouracil (DHU) concentration. Samples were collected between 8 and 11 AM to minimize the impact of circadian variations in DPD enzyme activity, and participants were instructed to fast between midnight and the time of sample collection [18, 19]. Samples were collected in EDTA tubes, placed on ice, and processed to plasma by centrifugation at 1500 g for ten minutes at 4 °C within 1 h of collection. Processed samples were stored at −80 °C until analysis.

Chemotherapy treatments with infusional 5-FU were delivered over 46 h via ambulatory infusion pump, as per institutional standard-of-care, with or without concomitant chemotherapeutic agents. All patients in the primary research cohort received the standard 2400 mg/m^2^ dose of 5-FU in cycle 1, and 5-FU dose adjustments after cycle 1 were made as per institutional standard of care. Pharmacokinetic blood samples were collected 1 to 5 h before the end of the 46 h 5-FU infusion in the first and second treatment cycles for measurement of 5-FU drug exposure. The 5 mL samples were collected in EDTA tubes, placed on ice, and processed to plasma by centrifugation at 1500 g for ten minutes at 4 °C within 1 h of collection. Processed samples were stored at −80 °C until analysis. If one of the pharmacokinetic samples was missed, a make-up sample could be collected in cycle 3.

### Measurement of plasma uracil, dihydrouracil and 5 FU concentrations

Measurements of plasma uracil, DHU, and 5-FU concentrations were conducted in the Clinical Pharmacology Shared Resource of the Dartmouth Cancer Center. Analyses of plasma uracil, DHU, and 5-FU concentration were conducted by ultra-high performance liquid chromatography and dual mass spectrometry, using a previously validated method with minor modifications [20]. 5-FU AUC was calculated by multiplying the measured steady state 5-FU concentration by the infusion duration, in hours [8].

### Pharmacogenomic analysis

Participants also provided a 5mL blood sample in EDTA tubes for pharmacogenomic analysis. DNA was extracted and stored at −80 °C until analysis. Prior to sequencing, DNA was fragmented and PCR-free library preparation was performed to generate unbiased, uniform coverage across the entire genome for next generation sequencing (NGS), starting from genomic DNA. Whole genome sequencing (WGS) was conducted for all subjects on the NovaSeq 6000 Illumina platform. WGS captured both coding and non-coding regions of the *DPYD* gene, including surrounding genomic context overlapping the locus encompassing the rs4294451 SNP of interest.

### Biostatistical analysis and sample size justification

We used descriptive statistics to report patient characteristics and characterize the distribution of plasma uracil concentration, rs4294451 genotype, and 5-FU AUC. For the primary analysis we estimated a longitudinal mixed-effects model where the dependent variable was the 5-FU AUC and the independent variables were the pretreatment plasma uracil concentration and the count of rs4294451 T-alleles (0, 1 [heterozygous], or 2 [homozygous]). Additional model covariates included cycle number (first or second), sex, serum creatinine (mg/dL), and 5-FU infusion dose (mg/m^2^), as well as a random effect for the patient (to account for clustering within patients across the first and second treatment cycles). We evaluated the primary study hypotheses by testing whether the beta coefficients for plasma uracil concentration and rs4294451 T-allele count were significantly different from zero, using a threshold of *p* < 0.05 to define statistical significance.

The planned sample size for the primary research cohort was 30 evaluable participants. Sample size calculations assumed a power of 80% to detect a correlation of 0.5 or greater between fasting plasma uracil concentration (standard deviation estimated from preliminary data as 2.1 ng/mL) and 5-FU AUC (standard deviation previously reported as 6.0 mg*h/L [12]).

## Results

The primary study cohort comprised 29 evaluable participants. The median age was 64 years (range 41–81) and nine participants (31%) were female. The most common cancer sites were colorectal (*n* = 15) and pancreatic (*n* = 10). Additional patient characteristics are shown in Table 1.

**Table 1:** Characteristics of patients receiving chemotherapy with a 46-hour 5-FU infusion

Characteristic	number (*n* = 29)	Percent (%)
Age group		
- 49 years and younger	5	17.2
- 50–59 years	5	17.2
- 60–69 years	13	44.8
- 70 years and older	6	20.7
Sex		
- Male	20	69.0
- Female	9	31.0
Race/ethnicity		
- White, non-Hispanic	29	100
Primary cancer site		
- Colorectal	15	51.7
- Pancreatic	10	34.5
- Gastroesophageal	2	6.9
- Other	2	6.9
Therapeutic intent		
- Curative	13	44.8
- Palliative	16	55.2
Chemotherapy regimen		
- 5-FU and leucovorin	2	6.9
- mFOLFOX6	17	58.6
- mFOLFIRINOX	10	34.5

### Plasma uracil concentration, *DPYD* rs4294451, and 5-FU AUC

The median fasting plasma uracil concentration was 10.20 ng/mL, with an interquartile range (IQR) of 7.54 to 12.30 ng/mL. The median DHU: uracil ratio was 7.35 (IQR 5.95, 9.23). Nine of 29 participants (31.0%) were carriers of the *DPYD* rs4294451 T-allele, including 7 heterozygous carriers (T/A) and 2 homozygous carriers (T/T).

**Fig. 1:**
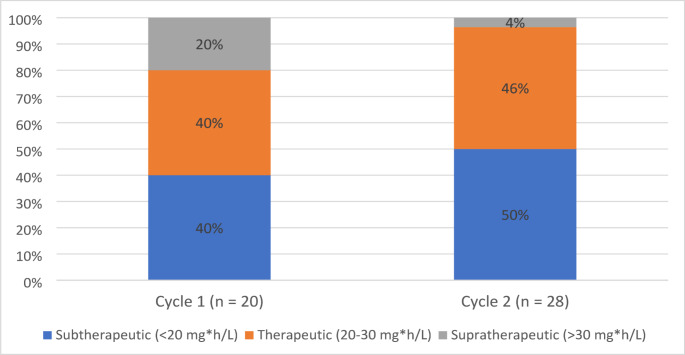
Categorical distribution of 5-FU AUC in cycles 1 and 2

5-FU drug exposure (measured as 5-FU AUC) was available for 20 subjects in cycle 1 and 28 subjects in cycle 2. The median 5-FU AUC was 22.4 mg*h/L (IQR 15.7, 28.4) in cycle 1, and 19.9 mg*h/L (IQR 16.0, 24.4) in cycle 2. The 5-FU AUC was within the therapeutic range of 20–30 mg*h/L for 8 of 20 patients (40%) evaluable in cycle 1 and 13 of 28 patients (46%) evaluable in cycle 2 (see Fig. 1).

**Fig. 2:**
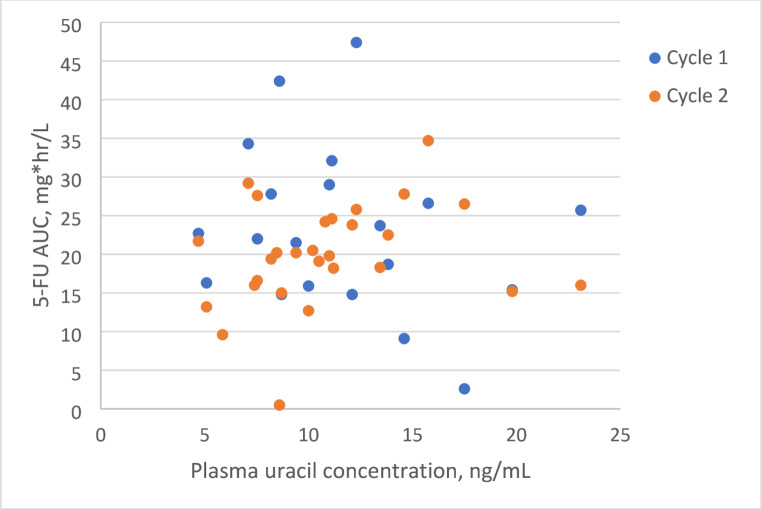
Scatter plot of pretreatment plasma uracil concentration and 5-FU AUC in cycles 1 and 2. Note: *R*^2^ is 0.05 for cycle 1 and 0.05 for cycle 2

In the multivariable mixed-effects model higher rs4294451 T-allele count (0, 1, or 2) was significantly associated with lower 5-FU AUC (beta = −4.0 mg*h/L per T-allele [95% CI −8.0, 0.0], *p* = 0.049). Male sex was also significantly associated with lower 5-FU AUC (beta = −7.5 mg*h/L [95% CI −13.8, −1.3], *p* = 0.021). However, fasting pretreatment plasma uracil concentration was not significantly associated with 5-FU AUC (beta = −0.16 mg*h/L per 1 ng/mL increase in plasma uracil concentration [95% CI −0.43, 0.75], *p* = 0.57). The estimated parameters of the mixed-effects model are shown in Table 2. We conducted an additional analysis by replacing the plasma uracil concentration in the multivariable mixed-effects model with the ratio of the dihydrouracil and uracil concentrations (DHU: U ratio). Like the plasma uracil concentration, the DHU: U ratio was not significantly associated with 5-FU AUC (*p* = 0.75). Figure 2 shows a scatterplot of plasma uracil concentration (x-axis) and 5-FU AUC (y-axis).

**Table 2:** Mixed-effects model of characteristics associated with 5-FU AUC

Covariate	Estimated beta coefficient	95% confidence interval	*p*-value
Intercept	−12.99	−56.88, 30.90	0.55
Cycle 2 (reference = cycle 1)	−2.72	−7.98, 2.53	0.29
Pretreatment plasma uracil concentration, ng/mL	0.16	−0.43, 0.75	0.57
rs4294451 T-allele count (0, 1, or 2)	−3.99	−7.98, −0.008	0.049
Male sex (reference = female)	−7.54	−13.78, −1.30	0.021
Serum creatinine, mg/dL	12.60	−4.07, 29.26	0.13
5-FU dose, mg/m^2^	0.013	−0.005, 0.031	0.14

We used the model parameters for fixed effects to calculate the predicted 5-FU AUC by sex and rs4294451 T-allele count. The base case for these predictions used a cycle 1 5-FU dose of 2400 mg/m^2^, plasma uracil concentration of 10.2 ng/mL (median value) and sex-specific median values for creatinine (0.65 mg/dL for females and 0.86 mg/dL for males). Among patients with the rs4294451 A/A genotype, male sex was associated with a predicted 17% reduction in 5-FU AUC, compared with female sex (predicted 5-FU AUC of 23.4 mg*h/L for males and 28.3 mg*h/L for females). Among males, the rs4294451 T/A genotype was associated with a 17% reduction in predicted 5-FU AUC, compared with the A/A genotype (predicted 5-FU AUC of 19.4 mg*h/L for a male with T/A genotype and of 23.4 mg*h/L for a male with A/A genotype).

### Early 5-FU related toxicity

Grade 3 or higher neutropenia during cycles 1–4 occurred in 9 of 29 subjects (31%). Thrombocytopenia of grade 3 or higher did not occur in any subjects. Toxicity-related reduction of the 5-FU infusion dose in cycles 2, 3, and/or 4 occurred in 8 subjects (28%). The composite outcome of early 5-FU-related toxicity (grade 3 neutropenia, grade 3 thrombocytopenia, or reduction of the 5-FU infusion dose) occurred in 12 of 29 subjects (41%). There was no significant association of the continuous pretreatment plasma uracil concentration with early 5-FU-related toxicity when evaluated by logistic regression (OR = 1.00, 95% CI 0.84–1.20). Early 5-FU-related toxicity occurred in one of eight patients (13%) with 5-FU AUC of less than 20 mg*h/L (subtherapeutic range) in cycle 1 and in 5 of 12 patients (42%) with 5-FU AUC of 20 mg*h/L or higher (therapeutic or supratherapeutic range) in cycle 1; this difference was not statistically significant (chi square *p* = 0.16). There was also no statistically significant association of the rs4294451 T-allele count with early 5-FU-related toxicity, though a clinically significant association was not excluded (OR = 0.52, 95% CI 0.14–2.03).

### Rare *DPYD* gene variants and plasma uracil concentration

Five participants were recruited to a secondary cohort of patients who were known carriers of rare deleterious *DPYD* gene variants, including three participants with the *DPYD* *2A variant (c.1905 + 1G >A), one with *13 (c.1679T >G), and one with HapB3 (c.1129–5923 C >G). Two additional carriers of the HapB3 variant were retrospectively identified in the primary study cohort. Three of the four participants with *2A or *13 (all in the secondary cohort) had a plasma uracil concentration of >16 ng/mL (above the threshold used to define DPD deficiency based on plasma uracil concentration [14]); the mean (median) pretreatment plasma uracil concentration in these four subjects was 18.0 (17.9) ng/mL. The three carriers of the *DPYD* HapB3 variant had pretreatment fasting plasma uracil concentrations of 6.0, 8.6, and 12.3 ng/mL, respectively. Neither of the two HapB3 carriers in the primary study cohort experienced early 5-FU-related toxicity despite receiving standard BSA-adjusted dosing of infusional 5-FU (2400 mg/m^2^).

Three of the 29 participants in the primary study cohort had uracil concentrations of >16 ng/mL, suggestive of DPD deficiency. The subject with the highest uracil concentration (23.1 ng/mL) had severe toxicity in cycle 1 (grade 3 neutropenia and grade 3 diarrhea), resulting in subsequent chemotherapy dose reduction. Exon-based sequencing of the *DPYD* gene identified a rare missense variant in this subject (c.2185G >A [p.A729T]). This variant has not been reported in the gnomAD population-based database; it is computationally predicted to have a deleterious effect on protein function (Revel in silico score = 0.742) [21]. The other two participants with plasma uracil concentration greater than 16 ng/mL did not have culprit *DPYD* gene variants; one developed grade 3 neutropenia in cycle 4 of chemotherapy, and the other tolerated standard-dose infusional 5-FU without early 5-FU-related toxicity.

## Discussion

This study was motivated by prior research showing that roughly half of all patients receiving standard doses of infusional 5-FU do not reach therapeutic-range drug exposure [10–12], and the primary study objective was to evaluate candidate biomarkers for subtherapeutic exposure to 5-FU. We evaluated 5-FU exposure over the first two cycles of chemotherapy in a cohort of 29 patients with gastrointestinal cancer. Similar to prior studies, we found subtherapeutic exposure to infusional 5-FU in nearly half of all treatment cycles evaluated.

We evaluated two biomarkers as candidate predictors of subtherapeutic 5-FU drug exposure—fasting pretreatment plasma uracil concentration and *DPYD* rs4294451 T-allele count. The study findings with respect to the rs4294451 locus were intriguing. This SNP was only recently identified as a novel biomarker of *DPYD* gene expression, and there has been little clinical study of this locus to date [16, 17]. The rs4294451 T-allele is relatively common, with a global minor allele frequency of 27% [16]. Our study is the first that we are aware of to find an association of rs4294451 T-allele count with lower 5-FU drug exposure (*p* = 0.049). The magnitude of the effect associated with the rs4294451 T-allele count appears to be clinically relevant; the 5-FU AUC for a heterozygous male carrier of this variant is predicted to be 17% lower than the 5-FU AUC for a male who is homozygous for the A/A genotype. Our finding of reduced 5-FU drug exposure in rs4294451 T-allele carriers is consistent with the prior finding that T-allele carriers had lower risk of toxicity with fluoropyrimidine-containing chemotherapy, as well as worse survival [17].

With regard to the pretreatment plasma uracil concentration, we did not find a significant association of this biomarker with the 5-FU AUC, and pretreatment plasma uracil concentration was also not associated with early 5-FU related toxicity. At least two other studies have similarly found that low plasma uracil concentration is not associated with outcomes of 5-FU-based therapy. Dolat and colleagues investigated the relationship between pretreatment plasma uracil concentration and 5-FU clearance, finding no correllation [22]. Kallee and colleagues evaluated the efficacy of fluorouracil-based chemotherapy in a heterogenous cohort of patients receiving 5-FU-containing chemotherapy. Analyses of progression-free and overall survival were stratified by pretreatment plasma uracil concentration (categorized as < 5 ng/mL, 5–16 ng/mL, or >16 ng/mL), and no between-group differences were identified [23]. We conclude that pretreatment plasma uracil concentration is not a promising biomarker for identifying patients at risk for subtherapeutic exposure to 5-FU.

In addition to the associations described above, our analysis also recapitulates the previously described association between sex and 5-FU AUC. Specifically, we found that male sex was associated with substantially lower 5-FU AUC, compared with female sex. This finding is consistent with several prior studies that have demonstrated a higher incidence of toxicity in women during 5-FU-based chemotherapy, compared with men [24–27]. Sex-based differences in 5-FU pharmacokinetics have also been described in prior research. For example, Milano and colleagues described significantly lower 5-FU clearance in females receiving 5-FU as a five-day infusion in 1992 [28], and Mueller and colleagues reported higher 5-FU AUC and increased toxicity among females receiving 5-FU by 46-hour infusion [29].

While our main analysis focused on identifying patients at risk for sub-therapeutic dosing of 5-FU, we also performed an exploratory analysis of the association of fasting pretreatment plasma uracil concentration with carriage of deleterious *DPYD* gene variants strongly linked with DPD deficiency. Of the four patients in our secondary study cohort with known *DPYD* variants in *2A or *13, three also had pretreatment plasma uracil concentrations of ≥ 16 ng/mL. Of the three patients who were carriers of the *DPYD* HapB3 variant, none had a pretreatment plasma uracil concentration of ≥ 16 ng/mL. Lastly, three patients had fasting pretreatment plasma uracil concentrations of ≥ 16 ng/mL without carrying a canonically deleterious mutation in *DPYD*. One of these patients carried a novel and likely deleterious *DPYD* gene variant (c.2185G > A [p.A729T]), while the other two patients did not have genetic or clinical evidence of DPD deficiency. These findings provide further context regarding the inconsistent relationship between *DPYD* genotype, fasting pretreatment plasma uracil concentration, and clinical outcomes of treatment with 5-FU-containing chemotherapy.

Prospective patient recruitment and biospecimen collection is a key strength of our study. Additionally, we followed rigorous procedures for collection of research blood samples. Specifically, blood samples for determination of the pretreatment plasma uracil concentration were collected in the morning, on ice, after an overnight fast; these procedures were followed to minimize pre-analytical variation that can affect the accuracy with which the plasma uracil concentration was measured [30]. The inferential strength of our study is limited by the modest sample size and by clinical heterogeneity of the study cohort with respect to primary cancer site, therapeutic intent, and other clinical and demographic characteristics.

In conclusion, our key finding is that rs4294451 T-allele count and male sex were independent predictors of reduced exposure to 5-FU chemotherapy. In conjunction with prior research showing that subtherapeutic exposure to 5-FU is associated with inferior treatment efficacy [7, 31], our research suggests that using the biomarkers of sex and rs4294451 T-allele count to adjust 5-FU chemotherapy dosing could lead to improved 5-FU exposure and, consequently, better cancer treatment outcomes. Future prospective studies of biomarker-guided dosing to optimize fluoropyrimidine chemotherapy exposure are warranted.

## Data Availability

The data that support the findings of this study are not openly available due to reasons of sensitivity. Research data are available from the corresponding author upon reasonable request. Data are stored in controlled access data storage at the Dartmouth Hitchcock Medical Center.

## Declarations

### Prior presentation

A subset of the findings described in this paper were previously presented at the 2024 ASCO Gastrointestinal Cancers Symposium (abstract 751, poster presentation).
